# Reactive oxygen species and p21^Waf1/Cip1^ are both essential for p53-mediated senescence of head and neck cancer cells

**DOI:** 10.1038/cddis.2015.44

**Published:** 2015-03-12

**Authors:** A L Fitzgerald, A A Osman, T-X Xie, A Patel, H Skinner, V Sandulache, J N Myers

**Affiliations:** 1 Graduate School of Biomedical Sciences, The University of Texas M. D. Anderson Cancer Center, Houston, TX, USA; 2 Department of Head and Neck Surgery, The University of Texas M. D. Anderson Cancer Center, Houston, TX, USA; 3 Department of Radiation Oncology, The University of Texas M. D. Anderson Cancer Center, Houston, TX; 4 Department of Otolaryngology-Head and Neck Surgery, Baylor College of Medicine, Houston, TX

## Abstract

Treatment of head and neck squamous cell carcinoma, HNSCC, often requires multimodal therapy, including radiation therapy. The efficacy of radiotherapy in controlling locoregional recurrence, the most frequent cause of death from HNSCC, is critically important for patient survival. One potential biomarker to determine radioresistance is *TP53* whose alterations are predictive of poor radiation response. DNA-damaging reactive oxygen species (ROS) are a by-product of ionizing radiation that lead to the activation of p53, transcription of p21^cip1/waf1^ and, in the case of wild-type *TP53* HNSCC cells, cause senescence. The expression of p21 and production of ROS have been associated with the induction of cellular senescence, but the intricate relationship between p21 and ROS and how they work together to induce senescence remains elusive. For the first time, we show that persistent exposure to low levels of the ROS, hydrogen peroxide, leads to the long-term expression of p21 in HNSCC cells with a partially functional *TP53*, resulting in senescence. We conclude that the level of ROS is crucial in initiating p53's transcription of p21 leading to senescence. It is p21's ability to sustain elevated levels of ROS, in turn, that allows for a long-term oxidative stress, and ensures an active p53–p21–ROS signaling loop. Our data offer a rationale to consider the use of either ROS inducing agents or therapies that increase p21 expression in combination with radiation as approaches in cancer therapy and emphasizes the importance of considering *TP53* status when selecting a patient's treatment options.

Head and neck squamous cell carcinoma (HNSCC) is the sixth leading cause of cancer worldwide, with an average of 50 000 new cases in the United States each year.^1^ Treatment of HNSCC often requires multimodal therapy, with external beam radiation therapy as a primary method of treatment for locoregional disease. As locoregional recurrence is the most frequent cause of death from HNSCC, the efficacy of radiotherapy in controlling this disease is critically important for patient survival. Some mechanisms of radioresistance have been proposed^2, 3^ yet relatively few biomarkers of radiosensitivity have been defined.

*TP53* has been shown to be the most common somatically mutated gene in HNSCC,^4^ with a majority of the mutations causing a disruption in the ability of the protein to transcribe genes important in regulating the cell's response to DNA damage. The functional status of p53 has been shown to be a significant predictor of response to chemotherapy.^5, 6^ Many prognostic parameters have been used to evaluate p53's functional status in a tumor, such as: location-based classifications,^7, 8, 9, 10^ type of amino-acid change,^11^ and posttranslational modifications.^12, 13, 14, 15^ To evaluate the impact of the *TP53* missense mutations used for this study, we have developed the Evolutionary Action scoring system.^16^ This system is a combination of two components: (1) the Evolutionary Trace approach, in which each sequence position is given a score that has been calculated to have a quantified impact on protein function based on evolutionary divergence data^17^ and (2) the type of amino-acid change at that position.

Expression of the p21^waf1/cip1^ gene is transcriptionally regulated by the p53 protein and is an essential mediator of p53-induced cell cycle arrest.^18^ p21's role in senescence, a terminal state of differentiation in which cells are permanently and irreversibly arrested, has been established under various conditions.^19, 20, 21^ Depending on cell type and stimuli, p21 is influential in causing cell senescence by multiple mechanisms, such as: binding to PCNA, leading to inhibition of DNA synthesis,^22^ interfering with cyclin-dependent kinase complexes,^23, 24^ inhibiting phosphorylation of Rb and, consequently, causing the downregulation of many E2F-regulated genes, which are involved in controlling progression of the cell cycle and DNA replication.^25^ A cell in a senescence state is considered to be tumor suppressive in that it can prevent transformation of cells;^26^ in addition, it can be a pathway to chemotherapy- or radiotherapy-induced cell death.^27^

Reactive oxygen species (ROS) are produced as by-products of standard cellular oxidative processes and have also been shown to be phenotypic markers of senescence.^28^ Also, depending on the cellular stress and level of oxidative damage, elevated levels of ROS can generate different cellular responses such as senescence or apoptosis.^26, 29^ Increased expression of p21 has been shown to lead to increased production of ROS in fibrosarcoma, prostate and bladder cancer cells with subsequent senescence.^26^ These data suggest that an interaction between p21 and ROS is involved in mediating the DNA damage-induced senescence response, but the specific aspects of this interaction are dependent on cell type.

Both p21 and ROS have been shown to be involved in senescence and although the complex relationship between p21 expression and ROS production has been studied for many years, questions as to how p21 and ROS regulate one another's expression remain. We show here evidence of a signaling pathway where the level and duration of ROS introduced are the determining factors in the initiation of either wild-type or partially functional p53 to transcribe p21. The elevated expression of p21, in turn, sustains the level of elevated ROS, causing HNSCC cells to undergo cell death via a senescence-mediated pathway.

## Results

### Null and mutant *TP53* HNSCC cells are resistant to radiation-induced senescence when compared with HNSCC cells expressing wild-type *TP53*

**Figure 1:**
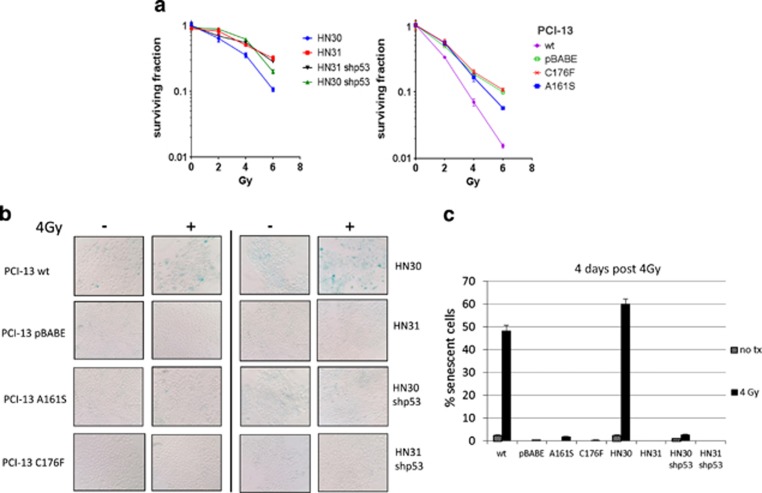
HNSCC cells with mutp53 exhibit resistance to radiation-induced senescence. (**a**) Clonogenic assay showing the differences in radiosensitivity among isogenic pair HN30 (wtp53) and HN31 (heterozygous mutant p53 alleles A161S and C176F), their p53 knockdown counterparts (shp53) and PCI-13 cells harboring either null or wt*TP53* or, A161S or C176F *TP53* mutations. (**b**) SA-*β*-gal staining of the cell lines described in **a** was performed 4 days after the cells were exposed to 4-Gy ionizing radiation. (**c**) Quantitation of SA-*β*-gal-positive senescent cells (those staining blue) from four randomly selected fields

To evaluate the radiosensitivity of HNSCC cells varying in p53 status, an isogenic pair of HNSCC cell lines, derived from the same patient differing only in their p53 status, HN30 (wild-type *TP53*, wtp53) and HN31 (*TP53* mutations, C176F and A161S) were used along with their p53 knockdown counterparts (HN30 shp53 and HN31 shp53). Also, to investigate the partial functionality of the A161S mutation independent of the nonfunctional C176F mutation, the wtp53 gene was inserted into HNSCC PCI-13 cells (*TP53* null) and site-directed mutagenesis was used to express either the C176F or A161S mutation. Clonogenic assays showed that the 4-Gy surviving fraction in *TP53* null or mutant cells was significantly higher than in cells with wtp53 (Figure 1a). Being that senescence is the primary mechanism of radiation-induced cell death in HNSCC cells,^30^ a senescence-associated *β*-galactosidase (SA-*β*-gal) staining was performed and showed that 4 days after treatment with 4-Gy ionizing radiation, cells with wtp53 had a significantly higher SA-*β*-gal-positive staining (48–60%) compared with the SA-*β*-gal-positive staining in *TP53* null and all *TP53* mutant cells (less than 5% Figures 1b and c). These results indicate that cells expressing mutant forms of *TP53* or are null for *TP53* are relatively radioresistant when compared with isogenic cells expressing wtp53, and that this radioresistance was associated with a lack of radiation-induced senescence.

### Loss of p21^waf1/cif1^ leads to a significant decrease of radiation-induced senescence in *TP53* wild-type cells

**Figure 2:**
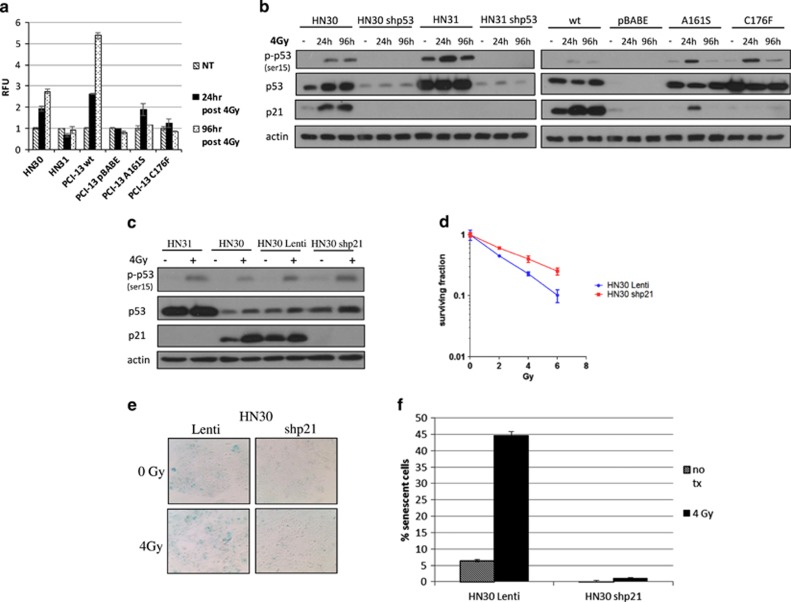
Long-term expression of p21^Waf1/Cip1^ is essential for radiation-induced senescence in HNSCC cells. (**a**) quantitative RT-PCR analysis of p21 mRNA expression in HNSCC cells treated with 4-Gy ionizing radiation and collected at indicated time points. Error bars represent S.D. of each sample performed in duplicate. (**b**) Western blot showing differences in p21 expression between wtp53 and mutp53 HNSCC cells when treated with 4-Gy ionizing radiation; protein lysates collected at the indicated time points. (**c**) Western blot results confirming lentiviral p21 knockdown in HN30 cells and their lack of response to ionizing radiation (**d**) Clonogenic assay showing resistance to ionizing radiation in HN30 cells with knockdown of p21 when compared with Lenti control. (**e**) SA-*β*-gal staining was performed 4 days after the cells were exposed to 4-Gy ionizing radiation. (**f**) Quantitation of SA-*β*-gal-positive senescent cells (those staining blue) from four randomly selected fields

To assess whether the lack of radiation-induced senescence seen in null and mutp53 HNSCC cells, relative to wtp53 HNSCC cells, was due to the loss of p53's transcriptional activity in the induction of p21, a protein well known to have an important role in mediating premature senescence, we measured the expression of the p21 gene by qRT-PCR after exposure to 4 Gy. As shown in Figure 2a, among the mutp53 bearing cells, only PCI-13 A161S had minimal, short-lived expression of the p21 gene compared with the enhanced, long-term expression seen in wtp53 cells. All other mutp53 or *TP53* null cell lines had no significant elevation in p21 mRNA. Western blot analysis demonstrated long-term expression of the p21 protein in wtp53 cells, with no expression in mutp53 and *TP53* null cells, but short-term expression in PCI-13 A161S cells (Figure 2b).

To further establish a relationship between p53, p21 and senescence in HNSCC cells, wtp53 HN30 cells, after stable knockdown of endogenous p21 with lentiviral shp21 vector (HN30 shp21; Figure 2c), were evaluated for their response to radiation. HN30 shp21 cells were found to be relatively resistant to ionizing radiation when compared with HN30 Lenti control cells (Figure 2d). Similar to null and mutp53 cells, this resistance was associated with a decrease in radiation-induced senescence (Figures 2e and f). Taken together, these findings indicate that p21 has a critical role in the therapeutic response of HNSCC cells to radiation.

### Transient expression of p21 in combination with ionizing radiation causes long-term p21 expression and elevated ROS, leading to a significant increase in senescence in HNSCC cells with mutp53

**Figure 3:**
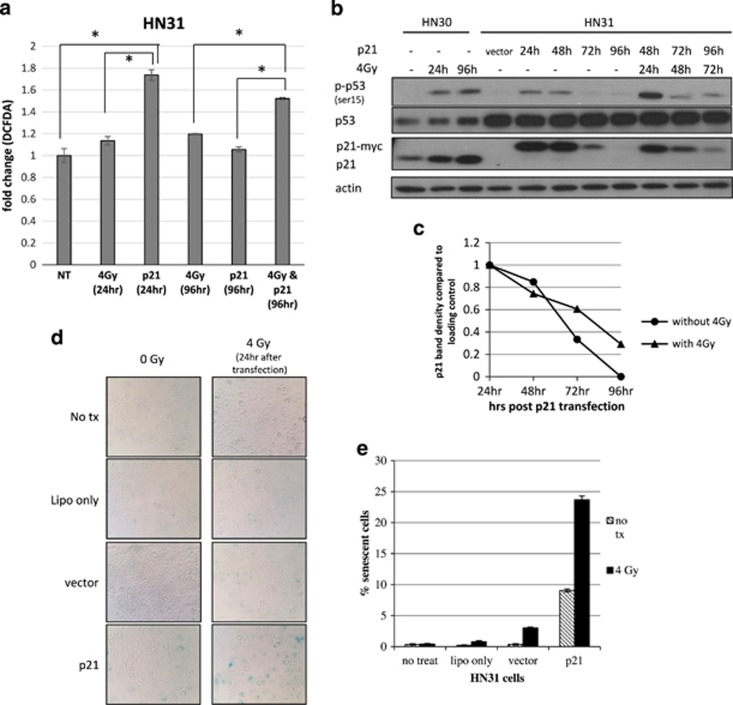
Transient expression of p21 in combination with ionizing radiation causes long-term p21 expression and elevated ROS, leading to a significant increase in senescence in HNSCC cells with mutp53. (**a**) Cells were collected at the indicated time points after treatment and then DCFDA staining, followed by flow cytometry was performed. (**b**) Western blot showing: endogenous p21 expression at early and late time points in HN30 cells exposed only to 4-Gy ionizing radiation and the extended stability of the p21 protein when HN31 cells are transiently transfected with p21 DNA in combination with 4-Gy ionizing radiation as compared with transient transfection alone. (**c**) Quantification of the stability of p21 between treatment conditions at each indicated time point when compared with loading control, actin. (**d**) Left column: HN31 cells were transiently transfected with the p21 DNA only; right column: 24 h after transfection, cells were irradiated with 4 Gy; SA-*β*-gal staining was performed 72 h later. (**e**) Quantitation of SA-*β*-gal-positive senescent cells (those staining blue) from four randomly selected fields. *P<0.05, by Student's *t*-test

Recent reports have shown that there is a strong correlation between p21, ROS and senescence with results varying by stimulus and cell type.^27, 28, 29^ As our mode of treatment for these HNSCC cells was ionizing radiation, we furthered our investigation into the p21–senescence relationship by measuring the effect that p21 overexpression would have on ROS. The use of 5-(and-6)-carboxy-20,70-dichlorofluorescein (DCFDA), a fluorescent marker of intracellular ROS, showed that the transient overexpression of Myc-tagged p21 in HN31 cells significantly increased the amount of ROS produced by cells at 24 h. Importantly, as the expression of the p21 protein went back to baseline at 96 h, so did the ROS levels (Figures 3a and b).

Noticing that the combination of p21 overexpression and radiation allowed for sustained elevation of ROS, along with long-term elevation of p21 and p-p53 protein levels, when compared with either p21 overexpression or radiation alone (Figures 3a–c), we wanted to determine whether this treatment would create sensitivity in HN31 cells, harboring the partially functional A161S p53 mutation, via the senescence mechanism seen in wtp53 cells. Figures 3d and e show that only in cells exposed to radiation and transient expression of p21 was there a significant increase in the percentage of senescent cells. These data suggest that p21 has a direct influence on ROS and that radiation treatment can enhance the sustained expression of p21, most likely through long-term, stress-induced phosphorylation of p53, and that this relationship is necessary for the induction of senescence.

### Long-term elevated ROS in cells with wtp53 is dependent on p21

**Figure 4:**
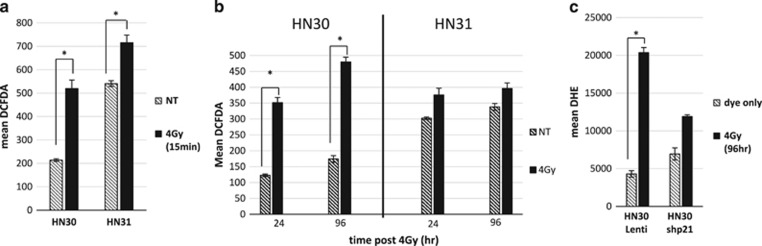
Long-term elevated ROS in cells with wtp53 is dependent on p21. (**a**) HN30 and HN31 cells were treated with 4-Gy ionizing radiation, while in suspension with DCFDA, then 15 min later, this initial burst of ROS was analyzed using flow cytometry. (**b**) Both HN30 and HN31 cells were exposed to 4 Gy, then were prepared for DCFDA staining and flow cytometry at indicated time points. (**c**) HN30 Lenti and HN30 shp21 cells were exposed to 4 Gy, then treated with the superoxide indicator, DHE, and collected for flow cytometry analysis at the late time point of 96 hrs. Error bars represent S.D. of each sample performed in triplicate. *P<0.05, by Student's *t*-test

Analysis of ROS levels revealed that HNSCC cells, regardless of p53 status, do have an initial burst of ROS 15 min after exposure to radiation, albeit at a much higher level in wtp53 cells (Figure 4a). Following the ROS levels for an extended time course showed that the initial ROS burst in wtp53 cells was not only sustained but became significantly elevated at the 96 h, when compared with the slight, nonsignificant increase seen in mutp53 cells (Figure 4b).

We then investigated the direct role of p21 on ROS sustainability. Being that HN30 Lenti and HN30 shp21 cells were GFP-tagged, dihydroethidium (DHE) was used as a superoxide indicator, as it exhibits fluorescent red within the nucleus upon oxidation. Figure 4c verified that cells with wtp53, but lacking p21 (HN30 shp21) do not have long-term elevated ROS levels post-ionizing radiation compared with the HN30 Lenti control. These findings suggest that the ability of wtp53 cells to undergo radiation-induced senescence is primarily due to p21's influence on the level of ROS produced by the cell.

### The use of antioxidants causes a significant decrease in the phosphorylation of p53, transcription of p21 and senescence

**Figure 5:**
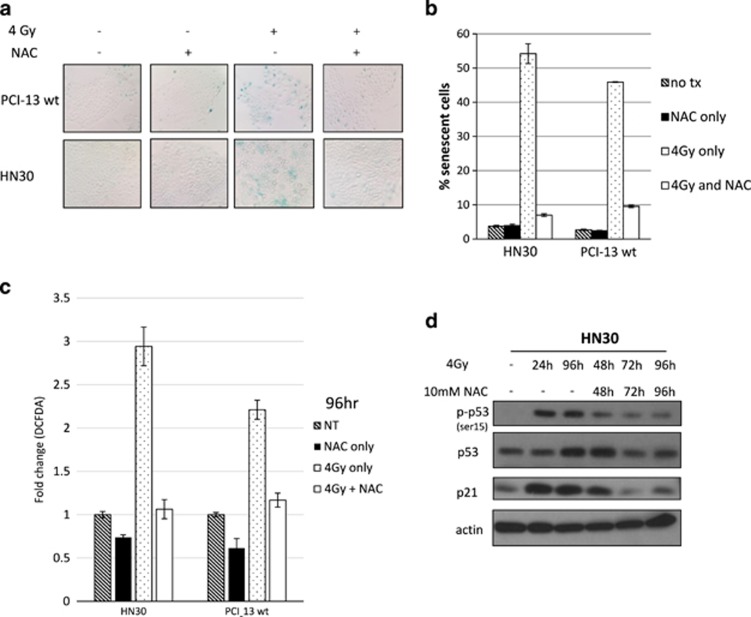
The use of antioxidants causes a significant decrease in senescence, long-term elevated ROS and p53 activity. (**a**) SA-*β*-gal performed 4 days after wtp53 cells were exposed to 4-Gy ionizing radiation with or without NAC treatment. (**b**) Quantitation of senescent cells (those staining blue) in four randomly selected fields. (**c**) DCFDA staining followed by flow cytometry of wtp53 cells performed 96 h after 4 Gy with or without NAC treatment. (**d**) Western blot displaying a time course of daily NAC treatment on p53 phosphorylation and p21 protein stability in HN30 cells after treatment with 4 Gy

To determine the impact of ROS in causing the senescence seen in wtp53 cells after treatment with radiation, HN30 and PCI-13 wtp53 cells were treated with the antioxidant, *N*-acetyl cysteine (NAC), 2 h before exposure to 4 Gy and every 24 h after, up to the 96 h time point at which the SA-*β*-gal staining was performed. NAC's suppression of intracellular ROS caused a significant decrease in senescence after radiation exposure when compared with cells not receiving NAC (Figures 5a and b). Figure 5c, confirms that daily treatment with NAC for 96 h inhibited ROS production after irradiation.

We were also interested to determine the effect that the inhibition of ROS would have on the induction of p53 and its transcriptional activation of p21. HN30 cells were irradiated at 4 Gy, then 24, 48 and 72 h later media was refreshed with NAC. We were able to see that at the later time points of 72 and 96 h, the ability of NAC to decrease ROS did indeed lead to decreased p53 phosphorylation and therefore a concomitant decrease in p21 (Figure 5d). These data support the notion that increased ROS levels lead to p21 induction through stress-induced activation of p53 and that this pathway leads to senescence.

### Short-term treatment with H_2_O_2_ dramatically elevates ROS with differences in p21 expression and senescence varying by *TP53* functional status

Noting that the difference in the baseline level of ROS in HN31 was two- to threefold higher than in HN30 (Figures 4a and b), we decided to investigate the possibility that cells with a partially functional *TP53* mutation may need a higher exposure to ROS, higher than the level produced using 4 Gy, in order to achieve a senescent state. Use of manganese (III) tetrakis (1-methyl-4-pyridyl) porphyrin pentachloride (MnTMPyP), a superoxide dismutase mimetic, determined that hydrogen peroxide (H_2_O_2_) was the most significant ROS contributing to senescence in wtp53 HNSCC cells because, while decreasing superoxide, MnTMPyP specifically creates the ROS intermediate, H_2_O_2_. Supplementary Figure 1A shows that by 96 h, HN30 cells, upon MnTMPyP treatment, do not completely suppress this elevation of H_2_O_2_, similar to when they are exposed to radiation. In addition, HNSCC cells treated with MnTMPyP alone exhibited a significant level of senescence, whereas cells treated with MnTMPyP and NAC allowed for an inhibition in the senescent phenotype (Supplementary Figures 1B and C).

**Figure 6:**
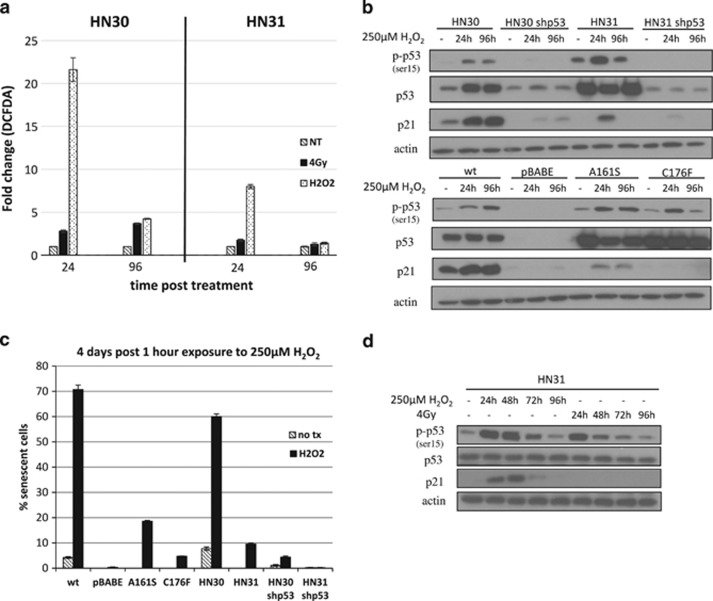
Short-term treatment with H_2_O_2_ dramatically elevates ROS with differences in p21 expression and senescence varying by *TP53* functional status. (**a**) DCFDA staining and flow cytometry of HN30 and HN31 cells treated with either 250 *μ*M H_2_O_2_ for 1 h or 4 Gy, with cells collected at the indicated time points after treatment. Error bars represent S.D. of each sample performed in triplicate. (**b**) Western blot of HNSCC cells treated with 250 *μ*M of H_2_O_2_ for 1  h with protein lysates collected at indicated time points. (**c**) Quantitation of SA-*β*-gal-positive senescent cells (those staining blue) from four randomly selected fields. (**d**) Western blot showing a time course of the difference in p21 protein stability and p53 activity in HN31 cells after treatment with either 250 uM for 1 hr or 4 Gy

In HN31 cells, direct treatment with 250 *μ*M of H_2_O_2_ for 1 h elevated the overall level of ROS to an eightfold increase at 24 h, compared with just a 23% increase after 4 Gy (Figure 6a). By 96 h, although both HN30 and HN31 cells were able to eliminate a majority of the intracellular ROS, the level of ROS seen in HN30 cells with H_2_O_2_ treatment was almost equivalent to the level seen with exposure to 4 Gy, the level that is necessary to induce senescence. However, HN31 cells were able to compensate for their massive ROS burst by reducing the ROS back to their baseline level. Collectively, these results provide evidence that HNSCC cells, regardless of their p53 status, do maintain redox capabilities; yet, cells with a mutation of *TP53* seem to have acquired the ability to reduce ROS to a level that will diminish the amount of permanent cell damage.

Still, with such a substantial increase of ROS after H_2_O_2_ treatment at 24 h in HN31 cells, we sought to determine if this would allow for any alterations to the cell. Figure 6b shows that treatment with H_2_O_2_ led to the short-term expression of p21 in HN31 cells and long-term expression of p21 not only in wtp53 cells but also in PCI-13 A161S, partially functional p53 cells. This translated into a significant increase in senescence for PCI-13 A161S cells and a notable increase in senescence for HN31 cells (Figure 6c).

To further examine the effect that H_2_O_2_ treatment would have on p53 activity, we looked at phospho-p53 and p21 protein levels over a 96 h time course in HN31 cells. We found that H_2_O_2_ treatment led to a p53-dependent increase in p21 protein levels for extended time points (returning to baseline levels at 96 h) when compared with the lack of increased p21 in irradiated cells (Figure 6d). These data support that it is the level of ROS produced from exogenous sources that leads to the induction of a partially functional *TP53*, leading to increases in p21 levels with an increase in senescence in these otherwise resistant HNSCC cells.

### Persistent exposure to a low dose of H_2_O_2_ results in ROS accumulation leading to long-term p53 phosphorylation, p21 transcription, and an increase in senescence in partially functional *TP53* HNSCC cells

**Figure 7:**
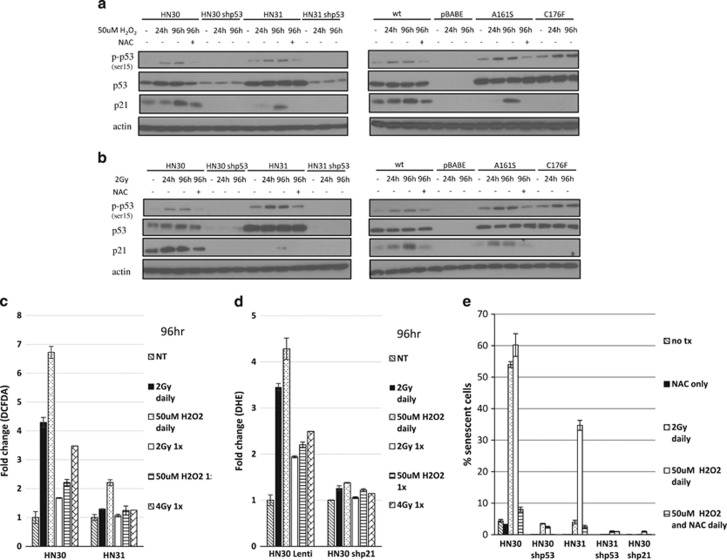
Persistent exposure to a low dose of H_2_O_2_ results in ROS accumulation leading to long-term p53 phosphorylation, p21 transcription and an increase in senescence in partially functional *TP53* HNSCC cells. Western blot showing differences in protein expression of p21 and phosphorylation of p53 at early and late time points in HNSCC cells after treatment with either (**a**) 50 *μ*M H_2_O_2_ for 1 h daily or (**b**) 2 Gy daily, with indicated cells lines also treated with NAC daily. C) DCFDA staining and flow cytometry performed 96 h after cells were treated with the conditions shown in **a** and **b** or, for comparison, with one treatment of either 2 Gy or 50 *μ*M H_2_O_2_. Error bars represent S.D. of each sample performed in triplicate. (**d**) Indicated cell lines were treated with conditions described in **c**, with DHE staining and flow cytometry performed at 96 h. Error bars represent S.D. of each sample performed in triplicate. (**e**) Quantitation of SA-*β*-gal-positive senescent cells (those staining blue) from four randomly selected fields

To further investigate the hypothesis that mutp53 cells were better adapted to reduce the burst of ROS produced by 4 Gy or 250 *μ*M H_2_O_2_, which may contribute to their relative radioresistance, we attempted to sensitize these cells by generating an environment of constant exposure to low doses of ROS by exposing them to 2 Gy or 50 *μ*M H_2_O_2_ every 24 h for 96 h. The protein level of p21 remained elevated in HN31 and PCI-13 A161S cells when treated with 50 *μ*M H_2_O_2_ daily. However, when these same cell lines were treated with 2 Gy daily, there was only long-term protein expression of p21 in the PCI-13 A161S cell line (Figures 7a and b). Also, cell lines treated with NAC daily showed a lack of p21 and p-p53 protein expression, again, confirming the significant impact of ROS and its role in the p53–p21–senescence pathway.

In HN31 cells, 50 *μ*M H_2_O_2_ daily treatment created a sustained twofold increase of ROS, whereas the level of ROS after treatment with 2 Gy daily was only elevated 29%, almost identical to one dose of 4 Gy at the same time point (Figure 7c). Also, the lack of ROS accumulation using these same treatment conditions in our HN30 shp21 cell line confirmed p21's role in regulating ROS (Figure 7d). Long-term activation of p53, p21, ROS expression and its contribution to senescence is shown in Figure 7e, in which there was a 35% increase in SA-*β*-gal staining in HN31 cells treated with 50 *μ*M H_2_O_2_ daily when compared with all other treatment types.

## Discussion

Given the prominent role of radiation therapy in the treatment of HNSCC, understanding the mechanisms of radiation resistance is of the utmost importance. Here we show that wtp53 HNSCC cells become terminally senescent after exposure to ionizing radiation, whereas mutp53 HNSCC are relatively radioresistant and avoid cell death. We also establish that the ability of HNSCC to senesce is due to the long-term expression of p21 whose transcription, under these conditions, is dependent on the functional status of *TP53*, and whose stability leads to elevation and prolonged expression of ROS. We also establish that HNSCC cells with a mutant *TP53* are relatively radioresistant because the level and duration of ROS produced from multiple doses of 2 Gy is not high enough to achieve a threshold necessary for cell senescence. But, with prolonged exposure to low doses of H_2_O_2_, an accumulation of ROS leads to senescence in cells with a partially functional *TP53*, by the same mechanism as seen in wtp53 cells.

We consistently observe that the radioresistant cell line, HN31 (with both A161S partially functional *TP53* mutation and C176F nonfunctional *TP53* mutation), has a two- to threefold higher basal level of ROS relative to its isogenic partner HN30 (wt*TP53*; Figures 4a and b). The high basal levels of ROS in HN31 suggest that their radioresistance may be due to an adaptation of these cells to a higher ROS environment, as others have shown that altered cellular antioxidant levels or mitochondrial production of ROS can influence the sensitivity of mutp53 cells to radiation.^26, 31, 32^ In a well-summarized review by Trachootham *et al.*,^33^ approaches to overcoming chemo and radioresistance in cancer treatment are currently being pursued by use of ROS modulating agents in a number of different cancer types.

p53 has been shown to be a transcription factor for genes other than p21 that can regulate senescence.^34^ To confirm the senescent phenotype was primarily dependent on p21, we used short hairpin RNA (shRNA) knockdown of p21 in cells with wtp53 and, indeed, after treatment with radiation, these cells showed significant resistance to senescence (Figures 2c–f). These results demonstrate that senescence in HNSCC cells is mediated primarily through p21, as opposed to other downstream p53 targets.

p21's influence on oxidative balance and the cell's response to modulation in the levels of p21 or ROS is varied, dependent on cell type, level and duration of p21 expression and the amount of ROS produced. How exactly p21 is able to regulate ROS is a burgeoning field of interest, yet remains to be completely understood. Chang *et al.*^35^ showed that p21 induction leads to an increased expression of a number of genes associated with extracellular matrix proteins, lysosomal enzymes and mitochondrial proteins, all of which have been associated with the induction of senescence. Other work has shown specific p21 interactions with pro-oxidant gene, PIG3, and antioxidant protein, Nrf2, in regulating the cells response to oxidative stress.^36, 37^

Although increasing p21 transcription causes HNSCC cells to senesce, some studies suggest that elevated p21 levels may have oncogenic effects.^38^ The complex pleiotropic effects of p21 could hinder the design of therapies focusing on p21 regulation. However, recent reports have shown that the induction of p21-dependent senescence using the NEDD8-activating enzyme inhibitor, MLN4924, can work as a mechanism of growth suppression in multiple cancer cell lines.^39^ In addition, histone deacetylase inhibitors can sensitize mutp53 HNSCC cells to radiation through p21 induction (A. Fitzgerald and J. Myers, unpublished observations).

In summary, we have identified a therapeutic signaling loop in which the level of elevated ROS is determinate when leading to the activation of partially functional p53, causing long-term expression of p21 that sustains ROS at these elevated levels, inducing HNSCC cells to undergo cell death through a senescence pathway. A decreased response to induction of p21 in response to radiation in mutp53 HNSCC cells, and therefore a reduced level of ROS production and sustainability, explains, in part, how they avoid senescence. This suggests that agents that can induce p21 and thereby prolong elevated ROS levels in mutp53 HNSCC in response to radiation could serve as effective radiosensitizers.

## Materials and methods

### Cell culture and constructs

HNSCC cell lines HN30 and HN31, originated in our lab, and PCI-13, acquired from Dr Jennifer Grandis (University of Pittsburg), were cultured in a 37 °C incubator in 5% CO_2_ atmosphere and maintained in Dulbecco modified Eagle medium that contained fetal bovine serum, penicillin/ streptomycin, glutamine, sodium pyruvate, nonessential amino acids and vitamins. Cells infected with GFP-tagged empty lentiviral vector (pLVTHM) or vector encoding an shRNA against p53 (pLVUH-shp53, Addgene Inc., Cambridge, MA, USA) were sorted using flow cytometry. Cells infected with GFP-tagged empty lentiviral vector (pGIPz) or vector encoding an shRNA against p21 (V3LHS_322234, Thermo Scientific, Pittsburgh, PA, USA) were selected using puromycin. Wild-type p53 construct was made by inserting wild-type p53 cDNA into the pBABE retoviral vector with A161S or C176F mutations generated within wild-type p53 vector using site-directed mutagenesis as described in Neskey *et al.*^16^ The wt, pBABE, A161S and C176F vectors were used to infect PCI-13 cells, which underwent selection with puromycin. All cell lines used in this study have been authenticated against the parental recipient cell line via short tandem repeat analysis.

### Clonogenic assay

HNSCC cells were seeded in six-well plates at predetermined densities for radiation doses ranging from 2 to 6 Gy, to allow for an equal number of ensuing colonies. The next day, cells were irradiated using a high-dose rate 137^Cs^ irradiator (4.5 Gy/min) and cultured for 8–10 days to allow for colony formation. Cells were then fixed in a 1.5% crystal violet/50% methanol solution. Colonies of more than 50 cells were counted and survival fraction was determined. All treatments were performed in triplicate.

### Senescence-associated *β*-gal staining

Senescence-associated *β*-gal staining was carried out according to the manufacturer's instructions (Cell Signaling, Danvers, MA, USA). In brief, HNSCC cells were plated in six-well plates and irradiated with 4 Gy on the next day. One hour before irradiation, NAC was added, and every 24 h refreshed with new media containing NAC. Cells were cultured normally for 96 h; they were then fixed for 15 min and stained overnight at 37 °C. Blue-staining cells were scored as senescent and reported as a percentage of all the cells observed per high-power field. Other treatments concerning hydrogen peroxide or multiple doses of radiation are described in Results.

### Immunoblotting

Cells were treated as indicated and washed once with cold PBS. Standard lysis buffer^40^ was then added to each plate and plates were incubated on ice. Cell lysates were then collected using a plastic scraper and centrifuged at 14 000 r.p.m. at 4 °C for 10 min. The supernatant was removed and total protein concentration was then calculated using the Bio-Rad Protein Assay (Bio-Rad, Hercules, CA, USA). Samples were prepared and immunoblot analysis was carried out as described previously.^41^ In brief, membranes were blocked in 5% milk for 1 h, then incubated overnight with anti-p53 DO-1 (Santa Cruz Biotechnology, Santa Cruz, CA, USA ), anti-phospho-p53 (Santa Cruz Biotechnology), anti-*β* actin (Cell Signaling) or anti-p21 (BD Pharmingen, San Jose, CA, USA) at 4°C. Membranes were washed with 0.1% Tween 20 in TBS and incubated for 1 h at room temperature with species-specific secondary antibody. Signal was generated using the Super-Signal West chemiluminescent system (Pierce Biotechnology, Rockford, IL, USA).

### ROS measurement

ROS levels were measured according to previously published protocols^42^ using CMH2-DCFDA or DHE dye (Invitrogen, Grand Island, NY, USA). In brief, cells were treated with irradiation, hydrogen peroxide or NAC as described in Results, or with 10 *μ*M MnTMPyP (Cayman Chemical, Ann Arbor, MI, USA) for 1 h, refreshed with media, then cells were collected at indicated time points by detachment with 0.5% trypsin, washed with Ca^−^/Mg^−^ PBS and resuspended in FACS tubes with PBS containing 5 *μ*M CMH2-DCFDA or DHE for 45 min. Fluorescence measurements were then conducted using a Beckman Coulter XL 4 color cytometer and the data were analyzed using Flow-Jo software.

### Transient transfection

The p21 cDNA was generously provided by Dr Guangan He and generation of the Myc-p21 vector is described in He *et al.*^43^ Myc-p21 or empty vector was transfected into HN31 cells with Lipofectamine2000 (Invitrogen), per the manufacturer's instructions. Cells were exposed to the transfection mixture containing 1 *μ*g of plasmid DNA for 5 h, washed and then replenished with normal media. At various time points, as described in Results, cells were collected either for FACS analysis after DCFDA preparation or for immunoblotting.

### RT-PCR

Isolation of total RNA of p21 in HNSCC cells collected at the indicated time points after 4 Gy irradiation was determined using quantitative reverse transcription PCR. Reverse transcription was completed with the use of the iScript cDNA synthesis kit (Bio-Rad), per manufacturer's instructions. The 7900HT Real Time PCR Systems (Applied Biosystems, Foster City, CA, USA) was used for quantitative real-time PCR, with Power SYBR Green PCR Master Mix (Applied Biosystems) using the following primers: p21 forward 5'-CGCTAATGGCGGGCTG-3′, reverse 5′-CGGTGACAAAGTCGAAGTTCC-3', GAPDH forward 5′-TGATGGTACATGACAAGGTGC-3′, GAPDH reverse 5′-ACAGTCCATGCCATCACTGC-3′. Duplicate samples were used, with the GAPDH gene used as an internal control.

## Abbreviations

HNSCC: head and neck squamous cell carcinoma
ROS: reactive oxygen species
shRNA: short hairpin RNA
NAC: *N*-acetyl cysteine
DCFDA: 5-(and-6)-carboxy-20,70-dichlorofluorescein
DHE: dihydroethidium
MnTMPyP: manganese (III) tetrakis (1-methyl-4-pyridyl) porphyrin pentachloride
SA-*β*-gal: senescence-associated *β*-gal
wtp53: wild-type *TP53*
mutp53: mutant *TP53*

## References

[bib1] Jemal A Bray F Center MM Ferlay J Ward E Forman D Global cancer statistics CA Cancer J Clin 2011 61 69 90 21296855 10.3322/caac.20107

[bib2] Moeller BJ Yordy JS Williams MD Giri U Raju U Molkentine DP DNA repair biomarker profiling of head and neck cancer: Ku80 expression predicts locoregional failure and death following radiotherapy Clin Cancer Res 2011 17 2035 2043 21349997 10.1158/1078-0432.CCR-10-2641 PMC3092475

[bib3] Skvortsov S Jimenez CR Knol JC Eichberger P Schiestl B Debbage P Radioresistant head and neck squamous cell carcinoma cells: intracellular signaling, putative biomarkers for tumor recurrences and possible therapeutic targets Radiother Oncol 2011 101 177 182 21700351 10.1016/j.radonc.2011.05.067

[bib4] Agrawal N Frederick MJ Pickering CR Bettegowda C Chang K Li RJ Exome sequencing of head and neck squamous cell carcinoma reveals inactivating mutations in NOTCH1 Science 2011 333 1154 1157 21798897 10.1126/science.1206923 PMC3162986

[bib5] Perrone F Bossi P Cortelazzi B Locati L Quattrone P Pierotti MA TP53 mutations and pathologic complete response to neoadjuvant cisplatin and fluorouracil chemotherapy in resected oral cavity squamous cell carcinoma J Clin Oncol 2010 28 761 766 20048189 10.1200/JCO.2009.22.4170

[bib6] Slovackova J Smarda J Smardova J Roscovitine-induced apoptosis of H1299 cells depends on functional status of p53 Neoplasma 2012 59 606 612 22862161 10.4149/neo_2012_077

[bib7] Cabelguenne A Blons H de Waziers I Carnot F Houllier AM Soussi T p53 alterations predict tumor response to neoadjuvant chemotherapy in head and neck squamous cell carcinoma: a prospective series J Clin Oncol 2000 18 1465 1473 10735894 10.1200/JCO.2000.18.7.1465

[bib8] Cho Y Gorina S Jeffrey PD Pavletich NP Crystal structure of a p53 tumor suppressor-DNA complex: understanding tumorigenic mutations Science 1994 265 346 355 8023157 10.1126/science.8023157

[bib9] Erber R Conradt C Homann N Enders C Finckh M Dietz A TP53 DNA contact mutations are selectively associated with allelic loss and have a strong clinical impact in head and neck cancer Oncogene 1998 16 1671 1679 9582015 10.1038/sj.onc.1201690

[bib10] Poeta ML Manola J Goldwasser MA Forastiere A Benoit N Califano JA TP53 mutations and survival in squamous-cell carcinoma of the head and neck N Engl J Med 2007 357 2552 2561 18094376 10.1056/NEJMoa073770 PMC2263014

[bib11] Petitjean A Mathe E Kato S Ishioka C Tavtigian SV Hainaut P Impact of mutant p53 functional properties on TP53 mutation patterns and tumor phenotype: lessons from recent developments in the IARC TP53 database Hum Mutat 2007 28 622 629 17311302 10.1002/humu.20495

[bib12] Ashcroft M Taya Y Vousden KH Stress signals utilize multiple pathways to stabilize p53 Mol Cell Biol 2000 20 3224 3233 10757806 10.1128/mcb.20.9.3224-3233.2000 PMC85616

[bib13] Sakaguchi K Sakamoto H Lewis MS Anderson CW Erickson JW Appella E Phosphorylation of serine 392 stabilizes the tetramer formation of tumor suppressor protein p53 Biochemistry 1997 36 10117 10124 9254608 10.1021/bi970759w

[bib14] Shieh SY Ikeda M Taya Y Prives C DNA damage-induced phosphorylation of p53 alleviates inhibition by MDM2 Cell 1997 91 325 334 9363941 10.1016/s0092-8674(00)80416-x

[bib15] Vousden KH Lu X Live or let die: the cell's response to p53 Nat Rev Cancer 2002 2 594 604 12154352 10.1038/nrc864

[bib16] Neskey DM Osman AA Ow TJ Katsonis P MCconald T Hicks S Evolutionary Action score of TP53 coding variants (EAp53) identifies high risk mutations associated with decreased survival and increased development of distant metastases in head and neck cancer J Clin Oncol 2015e-pub ahead of print 29 January 2015. 10.1158/0008-5472.CAN-14-2735 PMC4383697 25634208

[bib17] Lichtarge O Bourne HR Cohen FE An evolutionary trace method defines binding surfaces common to protein families J Mol Biol 1996 257 342 358 8609628 10.1006/jmbi.1996.0167

[bib18] el-Deiry WS Tokino T Velculescu VE Levy DB Parsons R Trent JM WAF1, a potential mediator of p53 tumor suppression Cell 1993 75 817 825 8242752 10.1016/0092-8674(93)90500-p

[bib19] Brown JP Wei W Sedivy JM Bypass of senescence after disruption of p21CIP1/WAF1 gene in normal diploid human fibroblasts Science 1997 277 831 834 9242615 10.1126/science.277.5327.831

[bib20] Noda A Ning Y Venable SF Pereira-Smith OM Smith JR Cloning of senescent cell-derived inhibitors of DNA synthesis using an expression screen Exp Cell Res 1994 211 90 98 8125163 10.1006/excr.1994.1063

[bib21] Vogt M Haggblom C Yeargin J Christiansen-Weber T Haas M Independent induction of senescence by p16INK4a and p21CIP1 in spontaneously immortalized human fibroblasts Cell Growth Differ 1998 9 139 146 9486850

[bib22] Li R Waga S Hannon GJ Beach D Stillman B Differential effects by the p21 CDK inhibitor on PCNA-dependent DNA replication and repair Nature 1994 371 534 537 7935768 10.1038/371534a0

[bib23] Harper JW Adami GR Wei N Keyomarsi K Elledge SJ The p21 Cdk-interacting protein Cip1 is a potent inhibitor of G1 cyclin-dependent kinases Cell 1993 75 805 816 8242751 10.1016/0092-8674(93)90499-g

[bib24] Xiong Y Hannon GJ Zhang H Casso D Kobayashi R Beach D p21 is a universal inhibitor of cyclin kinases Nature 1993 366 701 704 8259214 10.1038/366701a0

[bib25] Nevins JR Toward an understanding of the functional complexity of the E2F and retinoblastoma families Cell Growth Differ 1998 9 585 593 9716176

[bib26] Masgras I Carrera S de Verdier PJ Brennan P Majid A Makhtar W Reactive oxygen species and mitochondrial sensitivity to oxidative stress determine induction of cancer cell death by p21 J Biol Chem 2012 287 9845 9854 22311974 10.1074/jbc.M111.250357 PMC3322987

[bib27] Gadhikar MA Sciuto MR Alves MV Pickering CR Osman AA Neskey DM Chk1/2 inhibition overcomes the cisplatin resistance of head and neck cancer cells secondary to the loss of functional p53 Mol Cancer Ther 2013 12 1860 1873 23839309 10.1158/1535-7163.MCT-13-0157 PMC3955083

[bib28] Chen Q Fischer A Reagan JD Yan LJ Ames BN Oxidative DNA damage and senescence of human diploid fibroblast cells Proc Natl Acad Sci USA 1995 92 4337 4341 7753808 10.1073/pnas.92.10.4337 PMC41939

[bib29] Barzilai A Yamamoto K DNA damage responses to oxidative stress DNA Repair 2004 3 1109 1115 15279799 10.1016/j.dnarep.2004.03.002

[bib30] Skinner HD Sandulache VC Ow TJ Meyn RE Yordy JS Beadle BM TP53 disruptive mutations lead to head and neck cancer treatment failure through inhibition of radiation-induced senescence Clin Cancer Res 2012 18 290 300 22090360 10.1158/1078-0432.CCR-11-2260 PMC3251726

[bib31] Ni Chonghaile T Sarosiek KA Vo TT Ryan JA Tammareddi A Moore Vdel G Pretreatment mitochondrial priming correlates with clinical response to cytotoxic chemotherapy Science 2011 334 1129 1133 22033517 10.1126/science.1206727 PMC3280949

[bib32] Sandulache VC Skinner HD Ow TJ Zhang A Xia X Luchak JM Individualizing antimetabolic treatment strategies for head and neck squamous cell carcinoma based on TP53 mutational status Cancer 2012 118 711 721 21720999 10.1002/cncr.26321 PMC3188683

[bib33] Trachootham D Alexandre J Huang P Targeting cancer cells by ROS-mediated mechanisms: a radical therapeutic approach Nat Rev Drug Discov 2009 8 579 591 19478820 10.1038/nrd2803

[bib34] Qian Y Chen X Tumor suppression by p53: making cells senescent Histol Histopathol 2010 25 515 526 20183804 10.14670/hh-25.515 PMC2841029

[bib35] Chang BD Watanabe K Broude EV Fang J Poole JC Kalinichenko TV Effects of p21Waf1/Cip1/Sdi1 on cellular gene expression: implications for carcinogenesis, senescence, and age-related diseases Proc Natl Acad Sci USA 2000 97 4291 4296 10760295 10.1073/pnas.97.8.4291 PMC18232

[bib36] Chen W Sun Z Wang XJ Jiang T Huang Z Fang D Direct interaction between Nrf2 and p21(Cip1/WAF1) upregulates the Nrf2-mediated antioxidant response Mol Cell 2009 34 663 673 19560419 10.1016/j.molcel.2009.04.029 PMC2714804

[bib37] Macip S Igarashi M Fang L Chen A Pan ZQ Lee SW Inhibition of p21-mediated ROS accumulation can rescue p21-induced senescence EMBO J 2002 21 2180 2188 11980715 10.1093/emboj/21.9.2180 PMC125979

[bib38] Chang BD Broude EV Fang J Kalinichenko TV Abdryashitov R Poole JC p21Waf1/Cip1/Sdi1-induced growth arrest is associated with depletion of mitosis-control proteins and leads to abnormal mitosis and endoreduplication in recovering cells Oncogene 2000 19 2165 2170 10815808 10.1038/sj.onc.1203573

[bib39] Jia L Li H Sun Y Induction of p21-dependent senescence by an NAE inhibitor, MLN4924, as a mechanism of growth suppression Neoplasia 2011 13 561 569 21677879 10.1593/neo.11420 PMC3114249

[bib40] Zhou G Xie TX Zhao M Jasser SA Younes MN Sano D Reciprocal negative regulation between S100A7/psoriasin and beta-catenin signaling plays an important role in tumor progression of squamous cell carcinoma of oral cavity Oncogene 2008 27 3527 3538 18223693 10.1038/sj.onc.1211015

[bib41] Yigitbasi OG Younes MN Doan D Jasser SA Schiff BA Bucana CD Tumor cell and endothelial cell therapy of oral cancer by dual tyrosine kinase receptor blockade Cancer Res 2004 64 7977 7984 15520205 10.1158/0008-5472.CAN-04-1477

[bib42] Eruslanov E Kusmartsev S Identification of ROS using oxidized DCFDA and flow-cytometry Methods Mol Biol 2010 594 57 72 20072909 10.1007/978-1-60761-411-1_4

[bib43] He G Kuang J Huang Z Koomen J Kobayashi R Khokhar AR Upregulation of p27 and its inhibition of CDK2/cyclin E activity following DNA damage by a novel platinum agent are dependent on the expression of p21 Br J Cancer 2006 95 1514 1524 17088910 10.1038/sj.bjc.6603448 PMC2360737

