# Supplementary material for: Reactive oxygen species and p21Waf1/Cip1 are both essential for p53-mediated senescence of head and neck cancer cells
Source: Cell Death Dis. 2015 Mar 12;6(3):e1678–. doi: 10.1038/cddis.2015.44 (PMC4385922; doi:10.1038/cddis.2015.44)
Supplement: Supplementary Figure [file cddis201544x1.pdf]

Reactive Oxygen Species and p21$^{Waf1/Cip1}$ are Both Essential for p53 Mediated Senescence of Head and Neck Cancer Cells

Authors: Alison L. Fitzgerald, PhD**,** Abdullah A. Osman, PhD, Tong-Xin Xie, PhD, Ameeta Patel, BS, Heath Skinner, MD, PhD, Vlad Sandulache, MD, PhD, Jeffrey N. Myers, MD, PhD

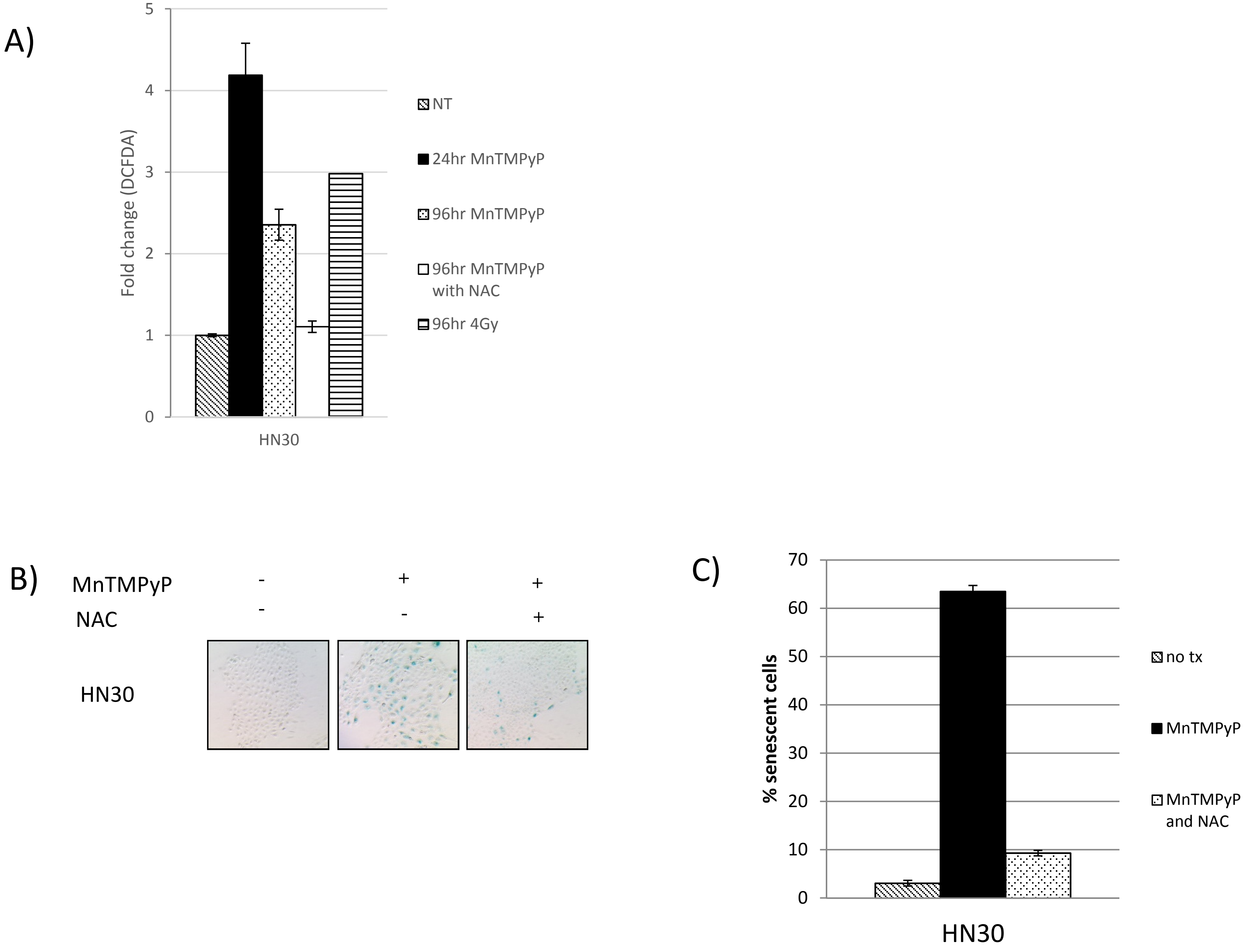

Supplementary Figure 1
