# Supplementary material for: Reactive oxygen species and p21Waf1/Cip1 are both essential for p53-mediated senescence of head and neck cancer cells
Source: Cell Death Dis. 2015 Mar 12;6(3):e1678–. doi: 10.1038/cddis.2015.44 (PMC4385922; doi:10.1038/cddis.2015.44)
Supplement: Supplementary Information [file cddis201544x2.docx]

**Supplementary Figure 1. H_2_O_2_ is the most significant ROS contributing to senescence in wtp53 HNSCC cells.** A) DCFDA staining and flow cytometry performed at indicated time points after cells were treated with MnTMPyP for 1 hr, with or without NAC daily, or 4Gy. B) SA-β-gal staining was performed 4 days after cells were exposed to MnTMPyP for 1 hr, with or without NAC daily. C) Quantitation of SA-β-gal positive senescent cells (those staining blue) from four randomly selected fields.
